# In-vitro gametogenesis on YouTube – Epistemological performances from Strasbourg and Los Angeles

**DOI:** 10.1016/j.rbms.2020.12.001

**Published:** 2020-12-13

**Authors:** Noémie Merleau-Ponty

**Affiliations:** Department of Sociology (ReproSoc), Univeristy of Cambridge, Cambridge, UK

**Keywords:** in-vitro gametogenesis, France, social sciences, USA, biology, assisted reproductive technology

## Abstract

YouTube hosts two records of interest for those interested in how human-stem-cell-derived gametes are made: one from the USA and one from France. Human-stem-cell-derived gametes, sometimes called ‘artificial gametes’ or ‘synthetic gametes’, are the result of in-vitro gametogenesis (IVG). IVG is a technology in the making that attempts to create oocytes and spermatozoa from embryonic cells or skin cells. This article presents some elements of these videos in written form, and asks what information is publicly available to ‘think with’, and what is not, when it comes to imagining the future of human reproduction. Focusing on the staging of science, this article argues that these videos represent ways of understanding and interrogating science, and display epistemological performances. The comparison is helpful to analyse how a shared global bioscientific authority is valued in these two locations, pointing at areas 'back stage' that the social sciences can illuminate.

## Introduction

YouTube hosts two records of interest for those interested in how human-stem-cell-derived gametes are made: 'Same-sex reproduction using stem-cell technology' (Natarajan, 2017) and 'La reproduction de demain: gametes artificiels [Tomorrow’s reproduction: artificial gametes]' (Forum Européen de Bioéthique, 2018). Human-stem-cell-derived gametes, sometimes called ‘artificial gametes’ or ‘synthetic gametes’, are the result of in-vitro gametogenesis (IVG), a technology in the making that attempts to create oocytes and spermatozoa from embryonic cells or skin cells. Two discussions about the reproductive future that this technology previews have been recorded: one in Los Angeles, USA and one in Strasbourg, France. Anyone with an internet connection and an understanding of English or French can access this well-known streaming platform and be given food for thought.

In this article, I will transcribe and describe some elements of these YouTube videos to pose a comparison that delineates how similar bioscientific papers are discussed in two different locations, showcasing different styles of imagining reproductive futures. Both events present their goal as an opportunity to open social dialogue between representatives of scientific knowledge and members of the public. What is publicly available to ‘think with’ and what is not? In this paper, I use ‘thinking with’ as an expression relating to knowledge-making practices, mixing ideas about human reproduction and the theories of science that accompany their expression or elaboration.

Using a technological framework of ‘culture’, I will argue that the ideas about human reproduction publicly available to think with are the result of epistemological performances that pose the authority of the biosciences, and silence social science knowledge to nurture public debates. By incorporating some knowledge from the social sciences into this text, I wish to reveal its ‘back staging’ and contribute to making it more visible.

## Materials and methods

### Materials: science on the social stage

The two videos that provoke this commentary are hosted by the widely known streaming platform YouTube. They were recorded at two different events: one in the USA and one in France.

The first video (Natarajan, 2017) was recorded on 8 June 2017 at the University of Southern California (USC) in Los Angeles as a TEDx event. The famous TED (Technology, Entertainment, Design) conferences showcase ‘ideas worth spreading’ (www.TED.com). The TEDx events are not official but benefit from the reputation of TED, whose website describes its local version as a way to nurture self-awareness and action in one’s community:

By organizing a TEDx event, you can create a unique gathering in your community that will unleash new ideas, inspire and inform. A TEDx event is a local gathering where live TED-like talks and performances are shared with the community (https://www.ted.com/participate/organize-a-local-tedx-event).

The second video was a round-table discussion recorded in Strasbourg on 3 February 2018 at the European Forum of Bioethics, a private association organizing a popular and well-attended annual event (Forum Européen de Bioéthique, 2018). The event is well known to many professionals involved in new biotechnologies for human reproduction and regeneration, and is attended by various citizens of all ages. The website of the European Forum of Bioethics insists on the ‘accessibility’ of bioethics questions to ‘all’ by ‘gathering European experts in front of the general public’ (https://www.forumeuropeendebioethique.eu/).

The status of these two videos is quite different. The video from Strasbourg draws from an institutionalized and ongoing public forum endowed with history and national authority. This forum is widely known in France and is attended each year by many specialists in the field, as well as many curious citizens. This video is a window into an example of how public spaces for discussions are valued when it comes to bioethics in France. One can also consider ‘États Généraux de la bioéthique’, held by the French state before revision of the French Bioethics Laws every 5 years, which brings together citizens and specialists in the areas at hand. On the other hand, the TEDxUSC event was a ‘one-off’ item in an ongoing public science stream, showcasing one individual and no questions and answers (Q&A). In the USA, there is no public space considered to institutionalize public bioethics, but many different discussions have spread across the USA and operate at different levels and through various types of media. Within the specificities of these two national contexts, both events share the common goal of putting a discussion about IVG into the public realm, valuing the social embedment of a novel reproductive biotechnology.

Interestingly, no empirical knowledge from the social sciences is represented in either of the videos. This article will incorporate some of this knowledge as a way to make the ‘back stage’ epistemological performance visible, and, hopefully, to bring forward some questions raised by this study.

### Analytical tools

#### Performance

This first analytical tool follows the notions of performance, theatre analogies, staging the subjunctive or the ‘bouillon cube’ of futurity and reproduction, pointing at the famous concepts of ‘social drama’ and ‘cultural performance’ (Turner, 1980: 149 and 160):

Social dramas occur within groups of persons who share values and interests and who have a real or alleged common history. The main actors are persons for whom the group has a high value priority. Most of us have what I call our ‘star’ group or groups to which we owe our deepest loyalty and whose fate is for us of the greatest personal concern.

The term ‘performance’ is, of course, derived from Old English *parfournir*, literally, ‘to furnish completely or thoroughly’. To perform is thus to bring something about, to consummate something, or to ‘carry out’ a play, order, or project. But in the carrying out, I hold, something new may be generated. The performance transforms itself. True, as I said, the rules may frame the performance, but the flow of action and interaction within that frame may conduce to hitherto unprecedented insights and even generate new symbols and meanings, which may be incorporated into subsequent performances. Traditional framings may have to be reframed – new bottles made for new wine.

#### Procreative imagination

This notion draws upon the work of Marit Melhuus (2007) to analyse how imaginations around procreation become projected worlds through which collective negotiations occur. These procreative imaginations become a means to discuss, regulate, limit or expand the development and applicative horizons of innovative technologies.

#### Operational relation

My own ethnography of ‘operational relations’ in in-vitro fertilization (IVF) and stem cell laboratories in India and France narrates how embryonic cell cultures are made ‘alive’ through localized uses and valuations of a global biological rationality. Operational relations provide a lens to study how biologists understand or wonder about the social value of their technical innovations and their connections beyond their bioscientific communities (Merleau-Ponty, 2018).

#### Recursivity

Sarah Franklin’s ethnography of stem cell biology (2013a) describes how basic science feeds into reproductive technology that feeds back into basic science that feeds back into reproductive technology. IVF was created in basic science laboratories, then introduced into the biomedical clinical realm, and has now enabled the field of stem cell basic science which enables the imaginative procreation that is IVG. However, recursivity is also epistemological as it points at the feedback loop within knowledge-making practices:

We can see in the stem cell lab, therefore, another interesting ‘intracultural’ comparison, namely how both anthropology and developmental biology have become more ‘recursively engaged’ with their equipment, and the empirical problem of its role in shaping experimental outcomes. This problem – the ‘agentic’ role of apparatus – is very familiar to any experimentalist. It is why scientific articles begin with lengthy descriptions of the precise materials and methods used in the experiment (Franklin, 2013b: 19).

## Results

### A certain style for scientific authority

In this section, the written description of certain parts of the videos will be used to display how the science of biology is ‘starred’ as grounding and enabling imaginative procreations. Similar biological articles are the global ground for scientific authority and local valuations of reproductive potentialities.

#### @TEDx: The queer swan of science!

At the time of this talk, Natasha Natarajan was a student majoring in human biology at USC while pursuing a Masters in stem cell biology and regenerative medicine. Natasha Natarajan starts her talk by standing on stage with a blank sheet of paper and says: ‘A stem cell is the blank page of the human body’. This analogy between writing and building a human body, between a blank page from which a novel is created and a stem cell from which a human body is developed, runs through all 16.46 min of the talk. This mundane analogy offers a tool to think about bioscientific novelties, as it connects well with another language analogy in biology: DNA as an alphabet with four letters through which a living organism is ‘written’. In this case, writing on a blank sheet of paper becomes the means to explain a rather difficult biotechnological notion – inducing stem cells from somatic cells:

Ten years ago, a brilliant scientist called Yamanaka asked: ‘well, what if we could go back, what if I wrote this [she shows the blank sheet of paper] in pencil instead of pen and there is a way to erase the words so that I would have a completely blank sheet of paper again? (…) Dr Yamanaka understood this one simple concept, that, just like us, as humans, cells are a product of their environment. So you can take any cell right now, put it in the same medium with these transcription factors that are normally found around a stem cell and it changes that cell back into a stem cell, or as we call ‘induced pluripotent stem cells’. It is like taking the last page out of your favourite novel, erasing it completely and writing a brand new story. In reality, biology is so much more fluid than we teach it to be (Natarajan, 2017: Youtube video 4.15 min).

**Figure 1:**
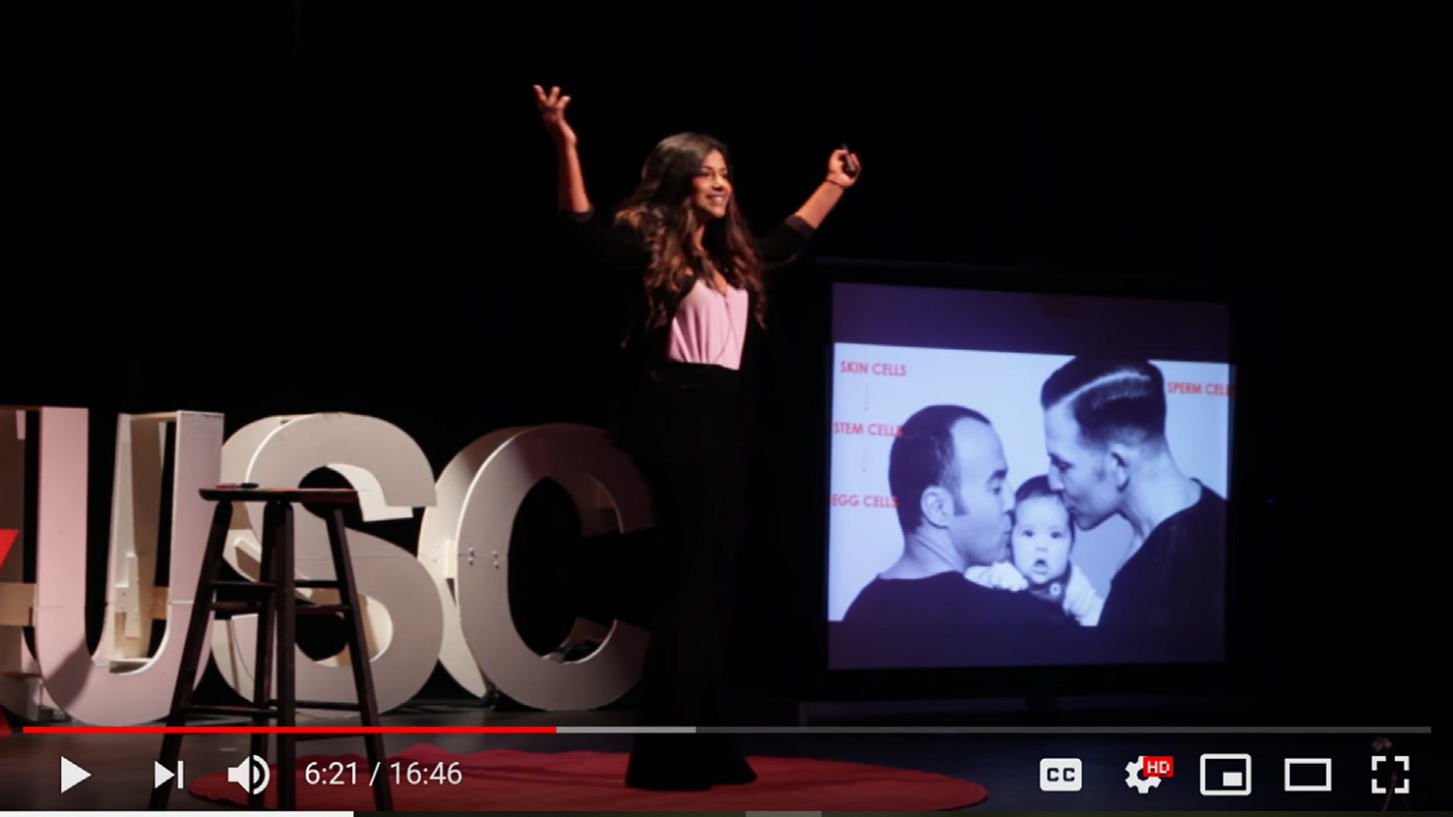
'Voilà!' says Natasha Natarajan, with a picture of two men and a baby in the background.

In this extended metaphor, not only is the fear of the blank page left at the margins, but writing itself becomes an occasion to think about fluid creativities associating cells, human beings and the wit of science at a very personal level. This creative mixture is then described as a way of giving science to the audience: ‘Now you have all the scientific tools you need to understand how same-sex reproduction works’. Indeed, now that we all have the scientific tools to understand same-sex reproduction, we hear about a male–male couple formed by Jim and Bob. Skin cells are taken from Jim and their information is erased to make a blank page on which information for egg cells will be written. These egg cells are then fertilized with Bob’s sperm cells, and the resulting embryo is transferred into the uterus of a surrogate (Figure 1). After this ‘wow’ moment, scientific articles appear on screen in the Powerpoint presentation. ‘Don’t get me wrong’, she says, ‘there are still a few more kinks to work out. But, this framework has been tested, supported’ and, she adds, ‘having that actually applied in our society’ is on the way.

Natasha Natarajan then asks the audience to imagine the use of blank paper in a way we have never imagined before, like making an origami swan. The use of origami to engage the public in stem cell biology has been described by Karen Jent (2017). While undertaking ethnography of a biology laboratory in Edinburgh, she also worked with the EuroStemCell Network and organized an exhibition called ‘Unfolding Organogenesis’. Origami figures were used to replicate how small organs could be grown in Petri dishes; this is how she describes the rationale behind such a project:

A further example of such affective and embodied connections to stem cell research is the ‘Unfolding Organogenesis’ exhibition that Alice and I collaboratively organized at the Edinburgh International Science Festival. […] How could the laboratory growth of organoid structures be addressed in a non-sensationalist way that did not play into the unfamiliarity and strangeness of organoid growth, but instead emphasized the challenges and routines that scientists face in their everyday work? Alice and I pondered these questions for months before settling on the idea of folding origami organs (Jent, 2017: 187).

**Figure 2:**
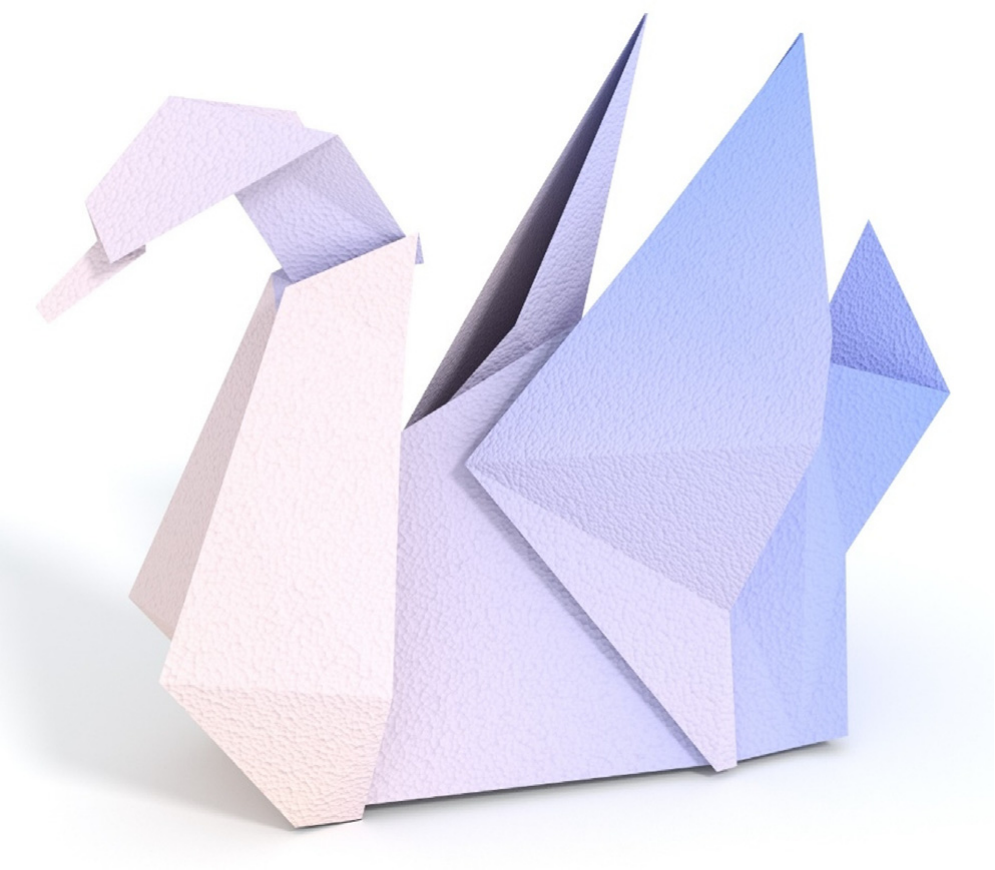
Origami figures can contribute to understanding pluripotency in a non-sensationalist way. Image courtesy of Ennelise Napoleoni-Bianco, Pixabay.

Origami figures are indeed quite helpful because a blank sheet of paper can be folded in numerous ways, showing how stem cells bear pluripotency and are also technologies for making new realities (Figure 2). The affective grammar of Natasha Natarajan’s talk is enthusiastic and plays with ‘wow, amazing’ feelings associated with science and its accessible tools.

In 2003–2004, three publications reported the production of primordial germ cells (an early stage of development before cells become ‘gametes’) in mouse models (Geijsen et al., 2004, Hubner et al., 2003, Toyooka et al., 2003); 8 years later, mouse pups were born in a Japanese laboratory following the fertilization of artificial gametes (Hayashi et al., 2011). This technique is also currently deployed with human cells in various laboratories across the world, and primordial germ cells have been produced in a British laboratory (Irie et al., 2015). Many technical challenges exist in building this ‘in-vitro system’ using human cells, with a biologist I interviewed in 2017 stating that ‘the question of future potentials, it’s quite long away’. When I asked a postdoctoral researcher, whom I invited to watch the video, for their thoughts, a calm eyebrow lifted: ‘she should come to the lab and see how it is done’, referring to the long, slow, repetitive work ending in more failures than successes. The following conversation revealed that this ‘American style’ of amazement was not the resercher’s ‘cup of tea’.

Nonetheless, ‘procreative imaginations’ (Melhuus, 2007) building on these articles are particularly vivid in both videos. The TEDx video focuses on same-sex reproduction, while the video from the European Forum of Bioethics in Strasbourg intertwines many different topics over 1 h and 44 min, from solo reproduction to cloning to eugenics and selection. Below, as for the US video, I will focus on how science is portrayed, enacted and circulated as procreative imaginations unfold in the Strasbourg media. Again, the authority of science is affirmed, although its enactment style differs strongly from the US video.

#### @Forum Européen de bioéthique: science and medicine know

This round-table discussion welcomes two biologists, one gynaecologist and one law practitioner, and is hosted by Professor Israel Nisand, the founder of the European Forum of Bioethics. The round-table discussion was in French, and all quotes have been translated by myself. The discussion is chaired by Samir Hamamah, Director of Studies at the National Institute for Science and Medical Research (INSERM) and Head of the Biology of Reproduction Department at the Public Research Hospital in Montpellier. Pierre Jouannet is a biologist and a member of the Ethics Committee of INSERM. Dr Catherine Rongières is a gynaecologist and coordinator of the Ethics Committee in Strasbourg. François Vialla is a jurist and Professor of Law at the University of Montpellier.

**Figure 3:**
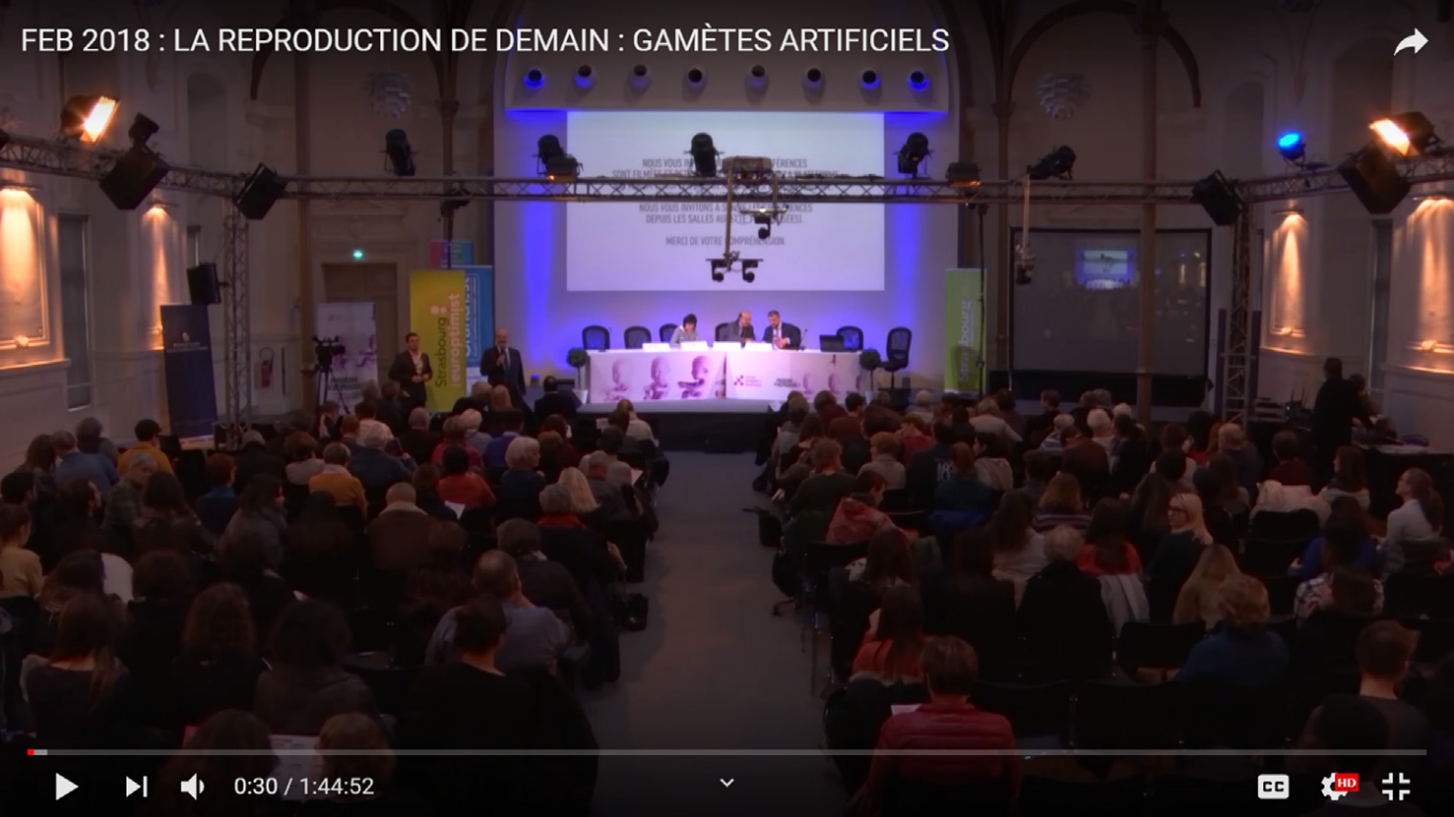
Professor Samir Hamamah, Catherine Rongières and Francois Vialla on stage.

Professor Samir Hamamah opens the short talks by describing the technology of IVG and ‘imagining’ three cases that he thinks are of bioethical importance: late maternity and ovulation after menopause; the end of gamete donation, anonymity and cross-border reproductive care; and solo reproduction. The latter consists of imagining how a single person could conceive alone using their existing gametes and generating complementary gametes. This would require the same protocol as described earlier: using the skin of the intended parent to induce pluripotency and differentiate the cells into either oocyte or spermatozoa. Next, Samir Hamamah opens the floor to other members of the round-table discussion: ‘Honour to women? Or rather the jurist? François’. The members of the round-table discussion are seated on a stage in front of a packed room (Figure 3). ‘The jurist’, François Vialla starts his contribution. He raises his left hand. A point is to be made: ‘*Medicus non sum*, I am not a doctor’. He then adds: ‘I authorize Professor Hamamah to hit my head if I say something stupid from the scientific point of view’. The joke works, and also makes a hierarchy of knowledge quite visible, recalling Dominique Memmi’s analysis of the first ethics committee in the 1980s as a gathering of ‘the ones who know’ (Memmi, 1996: 42). Vialla then summarizes the normative juridical landscape of France and the European Union, making a hypothesis of legal reasoning around artificial gametes. He insists on the relativity of the Law. Next, Pierre Jouannet associates solo reproduction with the end of men. Women would only generate female embryos as their cells are XX, so all the spermatozoa created from their cells would necessarily be X. He also mentions genome editing of the germline. Dr Catherine Rongières closes the talks by dismissing the topic of late motherhood introduced earlier by Professor Hamamah. She argues that her clinical experience makes her think that women generally do not wish to be mothers after their 40s. However, she has questions deriving from the scientific points that were made: What about the security of the technology for the offspring? What would it mean to have a society without men?

Many social scientists have worked on reproductive technology in France, publishing valuable knowledge on how families are conceived using gamete donation, or later in life or as a solo parent, either in France or by travelling abroad (Merchant, 2019). This knowledge is not performed onstage when the questions asked could be discussed using existing empirical data that bear strong similarities to ‘procreative imaginations’ around IVG.

Next, the Q&A starts, with another reminder of the strength of bioscientific rationale, in a context that seems tense. ‘We will try to discuss with the room’, stresses Professor Hamamah. ‘I recall to you what Professor François Jacob said: “There is nothing more dangerous than the certainty of being right”, so be moderate in your remarks please’.

One member of the public, a late adolescent, asks if ‘solo reproduction’ could be similar to ‘cloning’ as there would be ‘no genetic mixing’. The answer is no. Indeed, solo reproduction, although involving a single individual, would nonetheless entail genetic recombination as pairs of chromosomes would be re-organized through meiosis and fertilization. A woman asks what to think of ‘eugenics’ in that case. Would there be no more diseases? She appears to be calm and expresses genuine wonder. Hamamah says that he ‘has had enough’ of this debate as eugenics is linked to racism and Nazism. François Vialla distinguishes between a political wish to better the human race and a medical application at the demand of individual patients. Israel Nisand explains ‘why Samir was annoyed’, highlighting the difference between a positive and a negative form of eugenics: when one ‘chooses a handsome husband to have beautiful children, one does eugenics’.

Although radically different stylistically, both videos put bioscience and scientists on stage, playing the starring roles. The TEDx talk invites its audience to welcome bioscience as an amazing tool to fold and unfold fluid biologies, while the panel at the European Forum of Bioethics displays a sense of unease from the panelists and the public. These different styles stem from localized ways of evaluating the authority of biology, which have their own specificities, pointing at national contexts for bioethical discussions.

### Evaluating scientific authority

#### @TEDx: ‘you’ and ‘I’ and their private powers

This TEDx was organized by USC, a private university in Los Angeles focusing on individual skills, engagement with the world at large, and contribution to their community, as we can see from its website (https://admission.usc.edu/apply/our-admission-process/):

Our application process is designed to discover your individual story, so that we might see how you would take advantage of the many opportunities available at USC. […] USC students are unafraid to speak up in class to make others think or fight for a cause. They get involved by participating in student organizations and connecting with others. They seek to grow to their fullest potential, and they seek to serve others in the community along the way.

Scientific authority is mingled with assertiveness and powerful narratives of success. Participating in a TEDx surely looks like embodying UCS’ values of seizing opportunities to be engaged, involved and connected. This is clearly acknowledged by the speaker:

I first got interested in stem cells when I was just a 15-year-old high schooler, and my teacher gave us the opportunity to write about ‘em. Of course, in my mind, I was like ‘do I really wanna write a paper about a cell inside the stem of a plant, because, up until then, that’s all I thought a stem cell was. But 6 years later, with the college education from USC, I now know that it is so much more.

Natasha Natarajan is here to share what she had the opportunity to learn, in order to offer the members of the audience an opportunity to think about and engage with this topic. As scientific education is not separated from community engagement in this context, it is no surprise that she continues, saying how science is connected to moral, social, ethical, economic and political dynamics and that, of course, we all fear designer babies. However, this has nothing to do with the important topic that is same-sex reproduction, because, in that case, offspring would be born from a random sperm cell and a random egg cell. ‘We do not get to choose’. She then addresses the concern of a world populated solely by women. She opines that a few more women would not harm this world, right? For her, the real question is: Is it fair to prohibit lesbian women from giving birth to a few more women? The personal is political, and science can be a personal and political tool. Her sister is gay. ‘I love my sister. All I want for her in the world is to be able to have kids of her own.’ She understands stem cells ‘as an advocate for LGBTQ rights’, a way to seize the ‘opportunity to see the union of ‘science’ and ‘social advocacy’. From her own individual and very personal experience, she invites the audience to think as a society, an ‘us’ made of powerful individuals who can evaluate and govern science:

Where do we draw the line with ethical science? What is right? And what is wrong? […] The inescapable truth is that while scientists may wield the technology, only ‘you’ have the power to implement it.

The roles are made clear: science makes technologies with unprecedented reproductive potentials, and individuals can and should seize the opportunities to learn and find the means to govern them. Good science (Thompson, 2013) is certainly also enthusiastic and politically engaged. That said, to my knowledge, no gay or lesbian parents’ group has expressed interest in this particular technology. It is not because it could be biotechnically feasible for two men or two women to reproduce together that they would want to reproduce using this biological path. Before advocating for inclusivity and diversity on behalf of same-sex couples, it might be relevant to ask them what they think of this reproductive option.

Furthermore, this emphasis on biotechnological regulation may also obscure the consequences of biotechnological innovations in a neoliberal context. Charlotte Faircloth and Zeynep Gürtin’s introduction to a special issue on infertility and parenting notes that:

Paradoxically, while both ART [assisted reproductive technology] and parenting expertise aim to ‘assist’ reproductive agents in their conception and childrearing endeavours, they also generate new choices, burdens, responsibilities and accountabilities. The result, unsurprisingly, is increasing anxiety for parents and intending parents. In this moment of ‘anxious reproduction’, despite diligence and the use of multiple resources, reproductive agents often end up feeling overwhelmed, scrutinized and ‘not good enough’ parents (Faircloth and Gürtin, 2018: 995).

In this case, opportunities can become burdens and have difficult effects, pointing at the various struggles that can accompany assisted reproductive technology.

Speaking of difficulties, the evaluation of scientific authority at the panel at the European Forum of Bioethics displays mistrust from the public and anxiety from the scientists who stress how they are already governed in a political environment that they evaluate as polemical.

#### @Forum Européen de bioéthique: interlaced polemics

This forum can seem quite confused at first, but it helps if one understands the interlaced polemics at play. The heated discussion around definitions of cloning is polemic in the literal sense of the term, and it is also embedded in another polemic which points at the heated mutual evaluation of science and the public governance of science in France.

During the polemic around the similarity between solo reproduction and cloning, Bernard Baertschi, a bioethicist from the INSERM Ethics Committee, takes the microphone during the Q&A. He uses the term ‘cloning’ to stress the authority of the Law. ‘Cloning’, although feasible, is not performed in laboratories. Legal regulation can be very efficient. Pierre Jouannet reacts swiftly: ‘For once, I do not agree with you Bernard’. He clarifies what cloning means biologically and why solo reproduction using IVG would not be cloning. ‘We have to be cautious with the words we use’, he adds, narrating how the Foundation Jérôme Lejeune has used a misinterpretation of ‘cloning’ to fight a research project in court. Foundation Jérôme Lejeune is a private research funding body, inspired by Catholic values, especially those associating life with individuality and embryos with persons. The Foundation fights every research project involving human embryos and human embryonic stem cells in court.

The topic of the current evaluation of science runs through the panel in an escalation of expressed frustrations. From procreative imagination to current cultures of science, we hear from Pierre Jouannet that the actual legislation would not authorize research on IVG in France. Indeed, IVG would require the creation of embryos for research which is impossible in France, even if the National Committee for Ethics was favourable to such techniques in the 1980s. Catherine Rongières and Pierre Jouannet express regret that innovation is developed abroad and muzzled in France. Research risks should be taken, as they were by Steptoe and Edwards in the 1970s and by Belgian teams in the 1990s with intracytoplasmic sperm injection. Hamamah agrees and advocates for more research flexibility with ‘absolute transparency’. Defraissy adds that there is tension in France between the principle of precaution and research innovation. Professor Nisand has a question for the round-table: Is it not some kind of ‘scientific colonialism’ to let experiments be done abroad, let others take risks and then benefit from the successes after the clinical trials? ‘You are overreacting’, answers Pierre Jouannet. Although he thinks this question shows lack of moderation, he also stresses important differences in the rhythms of research between France and other, more flexible, countries. Cumbersome administrative oversight in France is slowing French scientific innovation significantly. Many researchers ‘lose courage completely’.

The last comment from the public is from a young girl: ‘You blame politics and legislation, but the population…we are maybe afraid of this research and of moving forward that direction, relating to the politically correct somehow’. Catherine Rongières has the last word. She does not address what it could mean for scientists and biomedical professionals to hear that the public is afraid. France needs to be inspired by what is done abroad ‘so that we could be put again in this infernal engine that is research’. Furthermore, the law ‘must adapt itself’ to transnational reproductive care in order to avoid the current ‘hypocritical’ system around egg donation, where French social security reimburses parts of treatments performed abroad.

These anxious expressions from the public and from the medicoscientific professionals seem to stem from a paradoxical situation of interlaced polemics. Medicoscientific professionals are in a position of authority over words to use and definitions to employ in a national context where they feel constantly threatened. After 1 h and 44 min of escalating polemics, viewers of this YouTube video will certainly have much to think about, as numerous complex scientific notions have been offered for discussion in a heated atmosphere.

The way I think about how both videos stage the authority of science for the discussion of reproductive imagination with a public audience is encapsulated in the label of ‘epistemological performance’.

## Discussion: epistemological performance

This paper has described the staging of a global bioscientific authority and the associated local evaluations through procreative imaginations. Indeed, let us remember that human IVG currently exists only in the imagination of humans. This imagination posits technological success derived from ongoing basic science.

This type of scientific performance is not an act of science, but enacts a certain way of understanding and interrogating science. It puts a theory of science on stage; it dramatizes an epistemology. Comparison is extremely helpful in this case because it helps localize, situate and contextualize epistemological performances, while bringing to the fore, again, the strength of biology as a global institution of knowledge (Franklin, 2000, Merleau-Ponty, 2018).

The shared epistemology performed through these YouTube videos stems from the idea that bioscience is sufficiently solid to allow for imaginations of the future and reproductive subjunctives to be discussed. This grounding characteristic of biology is also the consequence of the successful translation of IVF from basic science to biomedicine, and a more general contemporary emphasis valorizing translation. As exemplified in the Cooksey Report (2006) reviewing biological research in the UK and its potential for health, this emphasis highlights the idea that bioscience can and will produce new disruptive technologies. The latter will be applicable in the more or less distant future. Biocience is a star that stars our future.

Of course, localities also have a strong presence, providing a style to epistemological performances. This style is not only about underlining certain procreative imaginations over others, but also about evaluating and interrogating the very authority of biology. These ‘operational relations’ between global biology and local styles are performed differently depending on the contexts and the topics of their enactment. In this case, I have focused on how science itself is performed, valued and interrogated, and how it is to be seen through epistemologies. The TEDX video performs science as a learning opportunity to seize and to act on as a powerful individual citizen. The European Forum of Bioethics video performs science as an authoritative voice that can produce ‘fear’ in the public while scientists feel utterly constrained by legislation to the point of anger and deception.

Throughout this article, I have mentioned how empirical knowledge from the social sciences studying currently available reproductive technology is completely absent from the stage. Do same-sex couples wish to envision the use of artificial gametes to conceive? What about already-existing queer families, solo and late parents? Epistemological performance is also about what is not starred. The social sciences are well known for narrating back stage, enabling performances as they are made invisible. The anxiety expressed by a French member of the public at the end of the panel is displayed as a lack of trust in the good political intentions of bioscience. Mistrust in science is profoundly rooted in national histories. In France, eugenic politics during the Second World War have marked the science of biology with mistrust (Rabinow, 1999). Regarding the USA and stem cell research, Ruha Benjamin’s book *People’s Science: Bodies and Rights on the Stem Cell Frontier* (2013) addresses the complex history of public trust and scientific innovation directly. Ruha Benjamin shows, in great detail, how stem cell technologies in California were enabled through racial hierarchies, using some bodies in order to treat others. She also delineates how histories of malpractices against minorities can impede access later on, as contemporary patients can intensely mistrust the intentions of clinical trials, and decide not to participate in the development of new treatments when they are said to need adjustment based on the diversity of immune systems. Benjamin’s book is an excellent starting point to reflect on the ambiguous narrative of biology as a double-edged sword that essentializes while trying to diversify. It is also a great piece to think about the ways in which patients can be brought to the translational table while enabling their autonomy.

During the workshop from which this paper emerges, Faye Ginsburg noted the topic that is completely marginalized in these talks: the consequences for reproduction. How will care for the children after birth be organized? Marcia Inhorn noted that the future imaginary of IVG has already had profound consequences on procreative journeys, as some women cryopreserve their oocytes in the hope that their azoospermic partner may benefit from spermatogenesis in the future.

The social sciences remind us of the many complex mechanisms through which reproductive technology merges into the daily lives of families. All this knowledge ‘back stage’ of the biological scene is also part of contemporary ‘epistemological performances’. At a time when recursivity describes the loops from the laboratory to the hospital and back, through stronger awareness of the tools and mechanics that make stages as well as babies, turning the lights on the ‘back stage’ of such videos provides food for thought. Public tutelage is ‘DIY’ in the USA and more hierarchical in France, yet both assert the present and future hegemonic authority of the biosciences over reproductive biology. This hegemonic authority, although enacting intentions of public debate, also brings social polemics grounded in national histories of science. This article has advocated that epistemological performances could benefit from putting more empirical knowledge from the social sciences on stage in order to better understand what members of the public have to say about IVG.

*Declaration: The author reports no financial or commercial conflicts of interest*.

## References

[b0005] Benjamin R. (2013). People’s Science: Bodies and Rights on the Stem Cell Frontier.

[b0010] Cooksey, Sir D., 2006. A review of UK Health Research Funding. https://assets.publishing.service.gov.uk/government/uploads/system/uploads/attachment_data/file/228984/0118404881.pdf. 10.1136/bmj.39059.444120.80 PMC1702444 17170394

[b0020] Faircloth C.h., Gürtin Z.B. (2018). Fertile connections: Thinking across assisted reproductive technologies and parenting culture studies. Sociology.

[b0025] Forum Européen de Bioéthique, 2018. La reproduction de demain: gametes artificiels. https://www.youtube.com/watch?v=xHkVUSNs6Hw&t=873s.

[b0030] Franklin S. (2013). Biological Relatives: IVF, Stem Cells and the Future of Kinship.

[b0035] Franklin S. (2013). In vitro anthropos: New conception models for a recursive anthropology?. Cambridge Anthropol..

[b0040] Franklin, S. 2000. Life Itself. Global Nature and the Genetic Imaginary. In: Franklin, S., Lury, C., Stacey, J. (eds.), Global Nature, Global Culture, London, Royaume-Uni, Sage, pp. 188–227.

[b0045] Geijsen N., Horoschak M., Kim K., Grinau J., Eggan K., Daley G.Q. (2004). Derivation of embryonic germ cells and male gametes from embryonic stem cells. Nature.

[b9000] Hayashi K., Ohta H., Kurimoto K., Aramaki S., Saitou M. (2011). Reconstitution of the mouse germ cell specification pathway in culture by pluripotent stem cells. Cell.

[b0050] Hubner K., Fuhrmann G., Christenson L.K., Kehler J., Reinbold R., De La Fuente R., Wood J., Strauss J.F., Boiani M., Scholer H.R. (2003). Derivation of oocytes from mouse embryonic stem cells. Science.

[b0055] Jent, K., 2017. Making the Stem Cell Niche. PhD dissertation, University of Cambridge.

[b0060] Irie (2015). SOX17 is a critical specifier of human primordial germ cell fate. Cell.

[b0065] Melhuus, M. 2007. Procreative Imaginations: biotechnology, new reproductive technology and assisted conception in Norway. In: i Marianne E. Lien og Marit Melhuus (eds), Holding Worlds Together. Ethnographies of Knowing and Belonging. Berghahn Books, Oxford.

[b0070] Memmi, D., 1996. Les gardiens du corps: dix ans de magistère bioéthique, Paris, France, Éd. de l’École des hautes études en sciences sociales.

[b0075] Merleau-Ponty, N. 2018. Sélectionner des embryons humains. Une relation opératoire au sein de laboratoires de biologie de la reproduction en Inde et en France, L'Homme, n°225 (janvier-mars 2018), pp. 101–124. doi: 10.4000/lhomme.30724.

[b0080] Merchant J. (2019). Access to Assisted Reproductive Technologies. The Case of France and Belgium.

[b0105] Natarajan, N., 2017. Same-sex reproduction using stem-cell technology. TEDxUSC https://www.youtube.com/watch?v=k75DToh-ArU.

[b0085] Rabinow P. (1999). French DNA, Trouble in Purgatory.

[b0090] Thompson C.h. (2013). Good Science: the Ethical Choreography of Stem Cell Research.

[b0095] Turner V. (1980). Social dramas and stories about them. Crit. Inquiry.

[b0100] Toyooka Y., Tsunekawa N., Akasu R., Noce T. (2003). Embryonic stem cells can form germ cells in vitro. Proc. Natl. Acad. Sci. USA.

